# Surgical ablation in patients with atrial fibrillation and left ventricular dysfunction: A systematic review and meta-analysis

**DOI:** 10.1016/j.ijcha.2025.101648

**Published:** 2025-03-15

**Authors:** Leo Noanh Consoli, Eren Cetinel, Mir Wajid Majeed, Pawel Lajczak, Ilias Georgios Koziakas, Prajna Wijaya, Alexandros Apostolou, Raheel Ahmed, Konstantinos Perreas

**Affiliations:** a Federal University of Bahia, Bahia, Brazil; b San Raffaele University, Milan, Italy; c Government Medical College Srinagar, J&K, India; d Medical University of Silesia, Poland; e Onassis Cardiac Surgery Center, Greece; f Cardiac Surgery Department, Universitas Indonesia, Indonesia; g Hospital Clinic Zurich, Switzerland; h Cardiology Department, Royal Brompton Hospital, United Kingdom

**Keywords:** Surgical ablation, Atrial fibrillation, Left ventricular dysfunction, Heart failure

## Abstract

**Background:**

Atrial fibrillation (AF) and left ventricular dysfunction (LVD) are common conditions that often coexist, with about 25% of patients in NYHA classes I-II having AF. Efficacy and safety of surgical ablation (SA) in this population remains unclear.

**Methods:**

We aimed to perform a single-arm *meta*-analysis to assess the outcomes of standalone and concomitant SA in adult patients with AF and LVD. We searched PubMed, Scopus and the Cochrane Library. Endpoints of interest were maintenance of sinus rhythm, freedom from anti-arrhythmic drug (AAD) use, change in LVEF, 30-day mortality, 1 year mortality and major procedural complications. We calculated pooled proportions or means for binary and continuous endpoints, respectively, with a 95 % confidence interval (CI).

**Results:**

Ten observational studies comprising 863 patients (mean follow-up of 19 months) were included. At 1 year, SA resulted in a sinus rhythm rate of 83.9 % (95 % CI: 69.5–92.3); freedom from AAD use of 81.6 % (95 % CI: 64.7–91.7); and mortality of 5.77 (95 % CI:3.7–8.9). 30-day mortality was 2.16 % (95 % CI: 0.9–4.9); major complications 16.73 % (95 % CI: 12–23); and an improvement in LVEF of 12 % (95 % CI: 9–17).

**Conclusion:**

This *meta*-analysis found that SA appears to be an effective strategy to achieve sinus rhythm in patients with AF and LVD, with a pooled 30-day mortality of 2.2%. Comparative studies are warranted to evaluate the relative safety and efficacy of SA compared with other rhythm control strategies in this population.

## Highlights

• Surgical ablation is effective in patients with left ventricular dysfunction.
• 30-day mortality is comparable to what is seen in patients with normal left ventricular function.
• A reduced left ventricular ejection fraction should not be a contraindication for ablation.
• Care must be taken for major complications, particularly in patients at NYHA class IV.

## Graphical abstract

**Figure:**
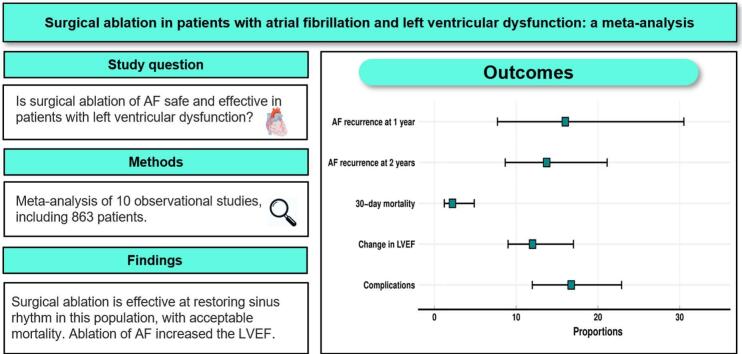

## 1 Introduction

Atrial fibrillation (AF) is the most common arrhythmia encountered in patients undergoing cardiac surgery [1] and is associated with increased morbidity and mortality, including higher risks of stroke, heart failure (HF), and prolonged hospital stay [2]. AF is highly prevalent in patients with left ventricular dysfunction (LVD), affecting an estimated 28.9 % of those with heart failure and reduced ejection fraction (HFrEF), 39.8 % with heart failure and mid-range ejection fraction and 45.2 % in patients with heart failure and preserved ejection fraction (HFpEF) [3]. Given the rising incidence of both conditions, cardiac surgeons frequently encounter such patients in their practice.

Surgical ablation (SA) of AF has been established as an effective strategy to restore sinus rhythm, often performed concomitantly with valve replacement, coronary artery bypass grafting, or as a stand-alone procedure [4]. However, its efficacy and safety in patients with LVD remains unclear, as these patients are underrepresented in most clinical trials of AF ablation. Considering that LVD is a risk factor during cardiopulmonary bypass, surgeons may avoid performing SA in these patients due to concerns that extending operative times could increase the risk of complications.

To address this gap in the literature, we conducted a *meta*-analysis to assess the outcomes of standalone and concomitant SA in patients with AF and LVD.

## 2 Methods

This systematic review and *meta*-analysis was prospectively registered in the International Prospective Register of Systematic Reviews (PROSPERO) under protocol CRD42024585744. This study was designed in accordance with the Cochrane Handbook for Systematic Reviews of Interventions [5] and adheres to the Preferred Reporting Items for Systematic Reviews and Meta-Analyses (PRISMA) guidelines [6].

### 2.1 Eligibility criteria

We restricted our analysis to studies meeting all of the following inclusion criteria: (1) clinical studies assessing standalone or concomitant SA of AF; (2) included adult patients with LVD; and (3) reported at least one of the following primary outcomes: maintenance of sinus rhythm after surgery, with and without anti-arrhythmic drug (AADs) use; and 30-day mortality; or secondary outcomes: change in LVEF pre and post-surgery; 1 year mortality; and major periprocedural complications (operative death; re-exploration for bleeding; mediastinitis; stroke; new dialysis; and pacemaker implantation). We excluded (1) studies with overlapping populations; (2) studies without an available full-text and (3) case reports, letters to the editor, comments or editorials. There were no restrictions concerning the date or language of publication.

### 2.2 Search strategy and data extraction

We systematically searched PubMed (MEDLINE), Embase and the Cochrane databases from inception to August 07, 2024. Our search strategy is available in the Supplementary appendix. We also searched within the references of all the articles included in our study. Article selection and data extraction were undertaken independently by two authors (L.C. and E.C.) using the prespecified criteria. Disagreements were resolved by a third author (P.W.). After removing duplicates, the authors screened titles and abstracts and assessed full-text articles for inclusion based on the inclusion and exclusion criteria. We extracted study characteristics, baseline patient data and any reported outcome of interest. Authors were contacted by email in case of missing data.

### 2.3 Quality assessment

We evaluated the methodological quality of each study using the methodological index for non-randomized studies (MINORS) tool [7]. We opted to use MINORS because our *meta*-analysis included mostly single-arm observational studies. Two independent reviewers (L.C. and P.W.) conducted this assessment, resolving any disagreements through consensus after a full-text article review.

### 2.4 Statistical analysis

One author (P.L.) conducted the statistical analysis using R software (version 4.1.1, R Foundation for Statistical Computing) via the meta package. We applied a random-effects model to all outcomes. To calculate pooled estimates and their 95 % confidence intervals (CIs), we used the generalized linear mixed model for proportions and the inverse variance method for means. We applied a natural logarithmic transformation to normalize the data before analysis. We evaluated heterogeneity among studies using the Baujat plot, chi-square (X^2^) and I-square (I^2^) tests with significance set at a p-value of less than 0.10 or I2 greater than 25 %. We examined the stability of the results through a leave-one-out sensitivity analysis. We assessed publication bias by a funnel plot analysis of point estimates based on study weights. For the outcome of change in LVEF, which represents the mean change between pre- and post-intervention means, we assumed a Pearson’s correlation coefficient of 0.5 to calculate the standard deviation (SD) of the change. To assess whether this assumption could bias our results, we performed a sensitivity analysis, recalculating the pooled mean of this outcome using different correlation coefficients ranging from 0.1 to 1.0. A subgroup analysis was performed comparing standalone and concomitant SA. We used a mixed effects *meta*-regression to assess the relationship between multiple covariates and the primary outcomes.

## 3 Results

### 3.1 Study selection and baseline characteristics

**Fig. 1:**
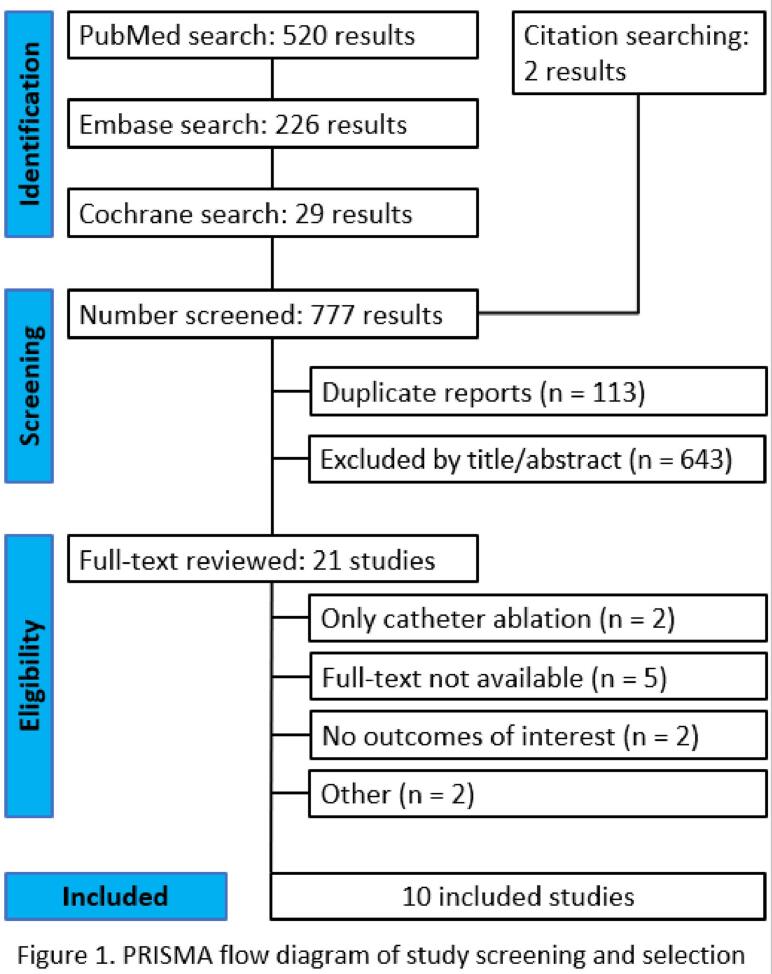
PRISMA flow-chart for the systematic review. Figure describing the sequential process of gathering and triaging studies for our review.

Our search strategy identified 777 studies, as detailed in Fig. 1. After removing duplicates and ineligible articles based on title/abstract review, 21 studies remained for full-text review based on inclusion and exclusion criteria. We included a total of 10 studies that met all inclusion criteria [[8], [9], [10], [11], [12], [13], [14], [15], [16], [17]], comprising a total of 863 patients. One study was excluded from the quantitative synthesis due to insufficient information for effect size analysis [16]; notably, this was the only study focusing on patients with HFpEF. Six studies [[8], [9], [10], [11], [12], [13]] were retrospective analyses while four were prospective cohorts [[14], [15], [16], [17]]. Four studies included a control group that either did not have HF or did not undergo SA [8,9,13,16].

The mean percentage of males was 66 % with a mean age of 61.1 years.. Mean follow-up duration ranged from was 41.8 months. Stand-alone SA was performed in 171 (19.8 %) patients, while the others underwent concomitant valve or coronary surgery. Regarding AF subtypes, the mean proportion of paroxysmal AF was 25.7 % to 74.3 % of chronic AF. Mean LVEF was 39.1 and the mean proportion of patients at NYHA classes III-IV was 53.1 %. The Cox-Mazeprocedure (CMP) was the most common lesion set, and radiofrequency was the most frequent energy source. Detailed information on patient characteristics is presented in Table 1 and further information on the surgical procedures can be found in Supplemental Table 1.

**Table 1:** Baseline characteristics of included studies.

**Study**	**Design**	**Number of patients, n**	**PAF, %**	**Male, %**	**Age, y**	**Type of concomitant surgery, n**	**LVEF**†**, %**	**Follow-up**†**, mo**	**NYHA III-IV, %**	**Lesion set – energy source**
Ad 2011	Prospective cohort	42	7	74	61.6	Valve: 19 CABG: 8 CABG + Valve: 7 Only ablation: 8	30	31.3	47.6	CMP – RF or CA
Grubitzsch 2007	Retrospective analysis	79	8.9	70.9	70	Valve:37 CABG: 27 CABG + Valve: 15	37	13	100	PVI – RF or MWA
Kim 2014	Retrospective analysis	77	2.6	58.4	51.6	Valve: 63 CABG + Valve: 14	36	66	58.4	CMP – CA or MWA
Pecha 2014	Retrospective analysis	59	41	28.8	68	Valve: 21 CABG: 15 CABG + Valve: 16 Other: 7	28	12	80	CMP (73 %) or PVI (27 %) – RF or CA
Ye 2023	Prospective cohort	247	NA	24.8	62.6	Valve: 247	>40	63.6	NA	CMP – RF
Pozzoli 2014	Retrospective analysis	39	0	92	51.3	Only ablation: 39	51.3	29.4	13	CMP – RF
Xie 2023	Prospective cohort	53	47.2	66	67	Only ablation: 53	62	39	34	PVI – RF
Stulak 2006	Retrospective analysis	37	37.8	92	55	Only ablation: 37	43.9	48	NA	CMP − RF
Rimac 2022	Retrospective analysis	196	65.8	79	67.8	Valve: 70 CABG: 38 CABG + Valve: 86 Other: 2	31.8	72	59.7	CMP (64 %) or PVI (36 %) – RF or CA
Adademir 2019	Prospective cohort	34	21	77	56	Only ablation: 34	32	44	32	CMP – RF

† mean or median; CABG: coronary artery bypass grafting; LVEF: left ventricle ejection fraction; NYHA: New York Heart Association; NA: not available; PAF: paroxysmal atrial fibrillation; CMP: cox-maze procedure; PVI: pulmonary vein isolation; RF: radiofrequency; CA: cryoablation: MWA: microwave ablation.

### 3.2 Quality assessment

Six studies [[10], [11], [12],^14^,^15^,^17^] were at moderate risk of bias and four at low risk [8,9,13,16], as detailed in Supplementary Table 3. The main concerns were lack of prospective sample size calculation and absence of blinding on the assessment of endpoints by the researchers. On funnel plot analysis, studies occupied an asymmetrical distribution according to weight, suggesting a risk of publication bias [Supplementary Fig. 17]. Nevertheless, due to high heterogeneity, we cannot conclude if the funnel plot asymmetry is truly caused by publication bias.

### 3.3 Primary endpoints

**Fig. 2:**
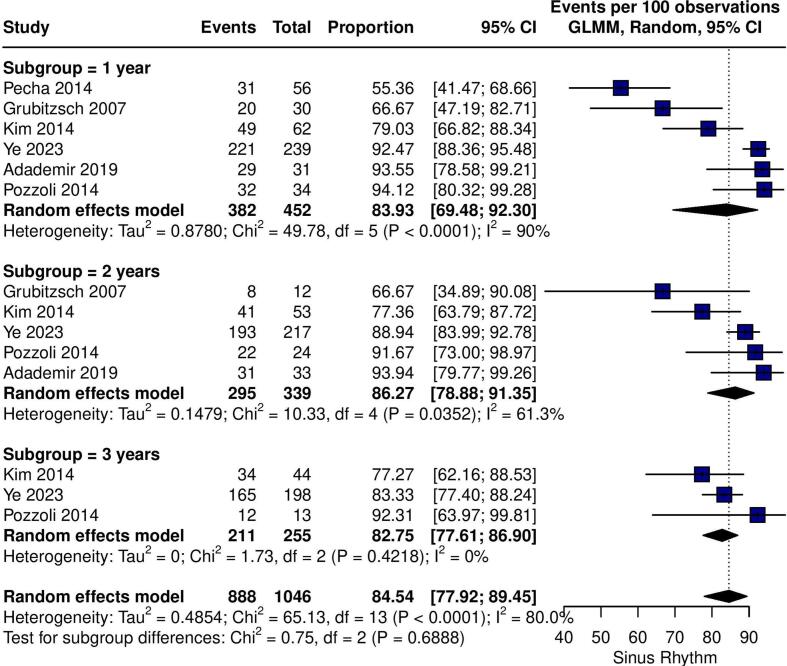
Forest plot for the outcome of maintenance of sinus rhythm. Forest plot showing the proportion of patients at sinus rhythm at 1, 2 and 3 years of follow-up. GLMM: grand linear mixed model. CI: confidence interval.

**Fig. 3:**
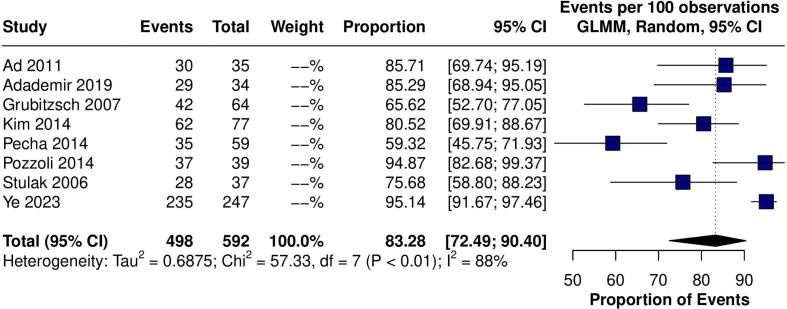
Forest plot for the outcome of 30-day mortality. Forest plot showing that in the pooled analysis the rate of 30-day mortality after surgery was 2.16%. GLMM: grand linear mixed model. CI: confidence interval.

At 1, 2 and 3 years, the pooled proportion of patients at sinus rhythm was 83.19 %, 85.30 % and 82.48 %, respectively (Fig. 2). At 1, 2 and 3 years, the pooled proportion of patients at sinus rhythm and free from AAD use was 81.04 %, 80.75 % and 80.07 %, respectively (Fig. 2). Regarding the main safety outcome of 30-day mortality, pooled rates were 2.16 % (95 % CI 0.95 – 4.87; I^2^ = 37 %; Tau^2^ = 0.65; Fig. 3).

### 3.4 Secondary endpoints

For the rate of complications pooled rates were 16.73 % (95 % CI, 11.97–22.90; I^2^ = 26 %; Tau^2^ = 0.1638; Supplementary Fig. 1). Pooled 1 year mortality was 5.77 % (95 % CI, 3.69–8.92; I^2^ = 30 %%; Tau^2^ = 0.03; Supplementary Fig. 3), while pooled mean improvement in LVEF was 12 % (95 % CI, 0.09–0.17; I^2^ = 96 %; Tau^2^ = 0.1169; Supplementary Fig. 4).

### 3.5 Sensitivity analysis

All results exhibited Tau^2^ heterogeneity values below 1, suggesting that the observed effects are consistent across studies. However, all I^2^ values were above 25 %. When individual studies were removed one at a time from all outcomes, no single study markedly altered the pooled results, indicating good stability [Supplementary Figs. 5–10]. However, in the leave-one-out analysis for 30-day mortality, excluding the study by Ye or Grubitzsch, reduced the I^2^ value from 37 % to 0 % indicating that these studies were the primary drivers of heterogeneity. Similarly, for the rate of complications, omission of the study by Pecha reduced the I^2^ from 26 % to 0 %. For the outcomes of 1 year mortality, sinus rhythm, and freedom from AAD use, the main contributors to heterogeneity were the studies by Grubitzsch and Ye, as identified by Baujat plots [Supplementary Figs. 11–16]. Regarding change in LVEF, heterogeneity was primarily driven by Stulak and Adademir. Sensitivity analysis of the correlation coefficient for this outcome [Supplementary Table 3] showed that varying assumptions did not significantly alter the pooled mean (0.1086 % to 0.1245 %) or I^2^ heterogeneity (93.1 % to 99.2 %).

### 3.6 Subgroup analysis

In the subgroup of standalone versus concomitant SA, outcomes were similar in both approaches (Supplementary Figs. 18–24). Standalone SA was associated with better rates of sinus rhythm at 1 year (93.8 % vs 77.2 %; p = 0.03; Supplementary Fig. 18), but not at subsequent follow-ups.

### 3.7 Meta-regression

We assessed the impact of patient age, publication year and percentage of patients with paroxysmal AF on the primary outcomes. Publication year was associated with an increase in the proportion of patients in sinus rhythm (p < 0.01; I^2^ = 65.5 %), indicating better rhythm control in more recent studies. Additionally, increased patient age was significantly associated with a higher risk of 30-day mortality and accounted for most of the variation in this outcome (p = 0.04; I^2^ = 23.4 %).

The proportion of patients with paroxysmal AF did not significantly influence the effect size of any outcome.

## 4 Discussion

In this systematic review and *meta*-analysis of 10 observational studies with 863 patients, we assessed the effects of SA in patients with AF and LVD. Our main findings were as follows: (1) the rates of 30-day mortality and periprocedural complications were 2.16 % and 16.73 %, respectively; (2) at 1 year of follow-up, sinus rhythm with and without AAD use was 83.19 % and 81.04 %, respectively and mortality was 5.77 %; (3) mean change in LVEF pre- and post-intervention was 12 %.

These findings suggest that SA is an effective strategy for restoring sinus rhythm in this population, with a relatively low mortality rate. A previous large *meta*-analysis of 23 RCTs in a general patient population found that SA resulted in a sinus rhythm rate of 70 % at 1-year follow-up, without increased risk of mortality compared to the no-ablation group [18]. Another single-arm *meta*-analysis of 36 studies on SA during mitral valve surgery reported a sinus rhythm rate of 83 % at 2 years of follow-up, with early and long-term mortality rates of 1.7 % and 7.5 %, respectively [19], indicating our results in patients with LVD are comparable to those in the broader population. The complication rate in our analysis was higher than reported in the literature, where other studies found major complication rates of 10–11 % [20,21]. We attribute this discrepancy to the high-risk nature of the population analyzed in our study. A recent systematic review of patients with AF and HF, concluded that in highly symptomatic patients refractory to medical therapy and catheter ablation, the CMP is a viable alternative to atrionodal ablation with permanent pacemaker implantation [22].

Many patients with chronic AF and reduced LVEF likely also have some component of tachycardia-induced cardiomyopathy (TIC), which can be reversible with arrhythmia control. Long-term maintenance of sinus rhythm is essential, as arrhythmia recurrence can quickly lead to symptomatic HF and worsened LV function. The observed improvement in LVEF in our study might reflect the inclusion of patients with TIC. While some improvement in LVEF is expected after surgical corrections for structural heart diseases, such as mitral valve repair [23] and coronary bypass [24] an improvement in the LVEF was still seen in the studies that assessed standalone ablation of AF. This finding is consistent with what is reported in catheter ablation in patients with HF, where a change in LVEF is seen even in the absence of additional surgical corrections [25].

When HF develops because of AF, guidelines recommend rhythm control via medical therapy, which has been demonstrated to be less effective than surgical and catheter-based interventions [26,27]. Currently, there is no clear indication on when to refer TIC patients for surgical ablation.

Among the studies reviewed, only Ye et. al [17] reported a decrease in LVEF during follow-up. Their cohort consisted of HFrEF patients with a recovered ejection fraction after optimal medical therapy, so the decline in LVEF may reflect a return to baseline function over time. Additionally, it's unlikely they included patients with TIC, as they excluded those with significant left ventricular remodeling, which might explain its significant contribution to the overall heterogeneity. Another major contributor to heterogeneity was the study by Grubitzsch [9]. Their population was the oldest (mean age 70 years) and most symptomatic, with a high comorbidity rate (all patients were NYHA class III-IV and 85 % had a prior myocardial infarction). These factors possibly contributed to the high mortality observed and its contribution to heterogeneity.

Regarding the studies reporting changes in symptom severity, there was a consistent decrease as measured by both NYHA class and EUROSCORE. Ad et al. [15] noted improvements in quality of life and functional capacity. Regarding HF-related hospitalizations, Ye reported a 5-year freedom rate of 65 % (95 % CI 58–72). At median follow-up of 66 months, Kim et al. examined a composite endpoint of death, HF or thromboembolic events, showing significantly better outcomes in the CMP group (HR = 0.28; 95 % CI 0.14–0.57; P = 0.0011). In contrast, Rimac found no significant difference in HF rehospitalization rates between the SA and the no-ablation groups (HR = 0.87; 95 % CI 0.62–1.22; P = 0.42). Rimac and Kim both compared HF patients undergoing Maze procedures versus no-ablation and found similar complication rates between the groups. Similarly, Xie did not observe a reduction in HF rehospitalization (HR = 0.69; 95 % CI 0.16–2.90; P = 0.61) but found that SA did not increase the rates of complications. Notably, Xie was the only study to include HFpEF patients. This study also stood out by employing a PVI lesion set via thoracoscopy. At 24 months, they reported an atrial arrhythmia recurrence rate of 39.5 % in the surgical group and 50.2 % in the medical management group (P = 0.32).

### 4.1 Study limitations

Our study had some limitations, including a lack of randomized data and variation in operative techniques among the studies. Analyzed articles were of moderate to high quality, but still had issues that are inherent to observational studies. Populations were also heterogeneous in terms of baseline LVEF, ratio of paroxysmal to persistent AF, comorbidities and symptom severity. Additionally, the scarcity of data in the literature about SA in patients with a severely reduced ejection fraction (<0.30) or diastolic dysfunction, limited our ability to generalize the results to these populations.

Despite these limitations, our current *meta*-analysis represents the most comprehensive synthesis of evidence on SA in this population to date. To account for differences among the included populations, we applied a random-effects model for all outcomes. Our results proved stable following a thorough sensitivity analysis, including leave-one-out and outlier analyses guided by Baujat plots.

## 5 Conclusion

In this systematic review and *meta*-analysis, our findings suggest that SA can be performed effectively in patients with AF and LVD, with a relatively low mortality rate. Furthermore, SA can lead to an improved LVEF and a reduction in HF symptoms. These findings can contribute to clinical decision-making and patient referral for cardiac surgery, but care must be taken regarding the considerable rate of complications associated with the procedure in this high-risk population. Additional comparative studies are needed to validate these results. Future research may aim to clarify the role of SA in patients with a severely reduced ejection fraction (<0.30) or with HFpEF.

## CRediT authorship contribution statement

**Leo Noanh Consoli:** Writing – review & editing, Writing – original draft, Methodology, Project administration, Investigation, Formal analysis, Data curation, Conceptualization. **Eren Cetinel:** Writing – review & editing, Writing – original draft, Data curation. **Mir Wajid Majeed:** Writing – review & editing, Conceptualization. **Pawel Lajczak:** Formal analysis, Data curation. **Ilias Georgios Koziakas:** Writing – review & editing, Data curation, Conceptualization. **Prajna Wijaya:** Formal analysis, Data curation. **Alexandros Apostolou:** Writing – review & editing, Data curation, Conceptualization. **Raheel Ahmed:** Writing – review & editing, Conceptualization. **Konstantinos Perreas:** Writing – review & editing, Supervision, Data curation, Conceptualization.

## Declaration of competing interest

The authors declare that they have no known competing financial interests or personal relationships that could have appeared to influence the work reported in this paper.
