# Supplementary material for: Surgical ablation in patients with atrial fibrillation and left ventricular dysfunction: A systematic review and meta-analysis
Source: Int J Cardiol Heart Vasc. 2025 Mar 15;58:101648. doi: 10.1016/j.ijcha.2025.101648 (PMC11952843; doi:10.1016/j.ijcha.2025.101648)
Supplement: Supplementary Data 1 [file mmc1.docx]

**Supplementary material**

**Table of contents**

**Appendix 1: Search strategy ................................................................................................. 2 Appendix 2: Additional forest plots……………...................................................................3 Appendix 3: Surgical characteristics....................................................................................6 Appendix 4: Sensitivity analysis …....................................................................................... 8 Appendix 5: Subgroup analysis …....................................................................................... 16** **Appendix 6: Risk of bias analysis.......................................................................................... 23**

**Appendix 1: Search strategy**

Available below is the search strategy we created for PubMed and modified for the other databases as was necessary using relevant terms and syntax. All references from selected studies were also manually retrieved for backwards snowballing.

**PubMed search strategy:**

("surgical ablation" OR "ablation surgery" OR "cox-maze" OR "maze surgery" OR "atrial fibrillation surgery") AND ("left ventricular failure" OR "left ventricular dysfunction" OR "heart failure") NOT ("catheter ablation"[tiab] OR "transcatheter"[tiab] OR "catheter"[tiab])

**Appendix 2: Additional forest plots**

**Supplementary Figure 1:**

**
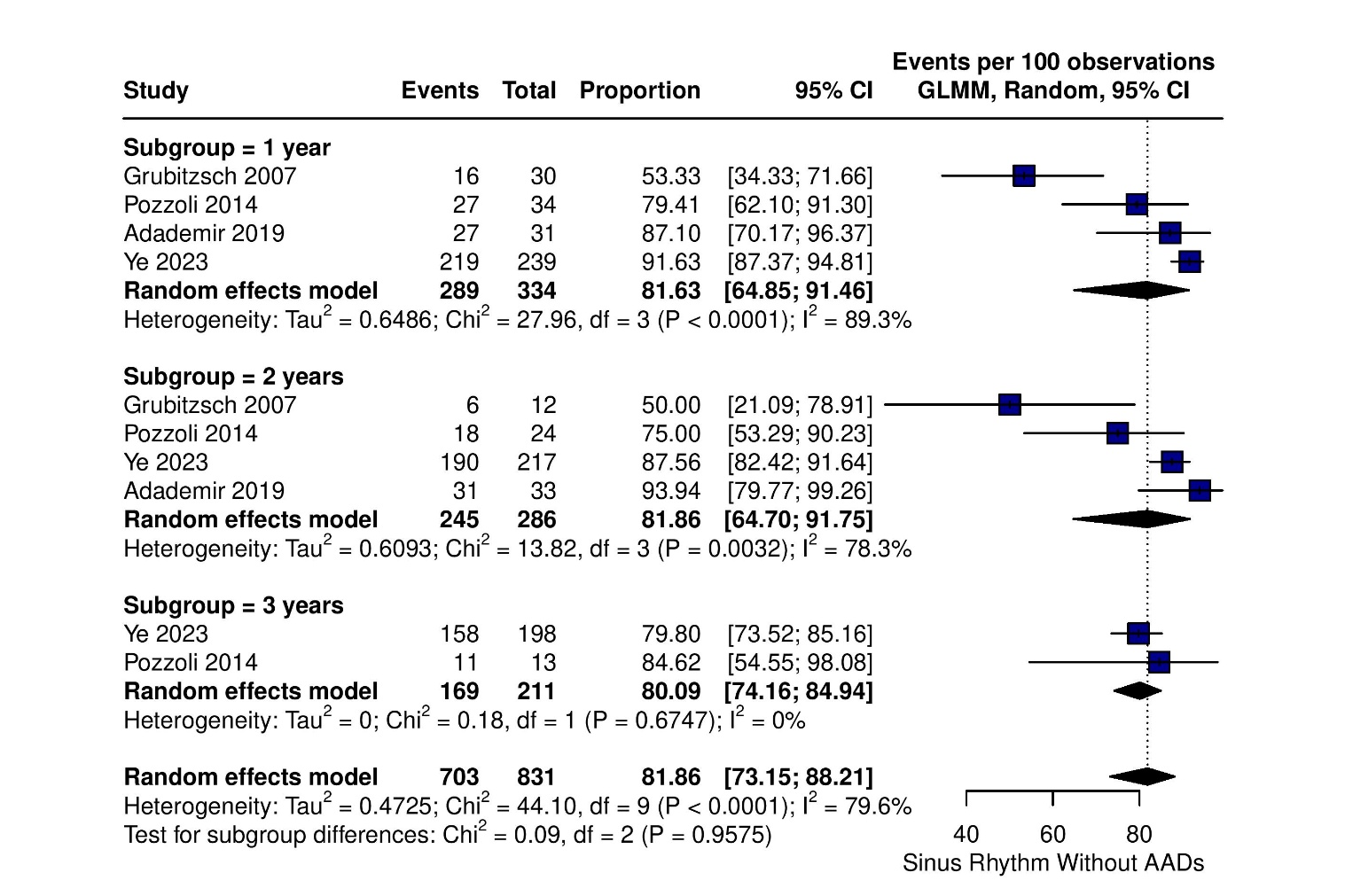
**

Freedom from anti-arrhythmic drug use, random effects model. Forest plot of the aggregate evidence showed that 81.63% of patients were at sinus rhythm without needing anti-arrhythmic drugs at 1 year of follow-up. CI: confidence interval. GLMM: generalized linear mixed-effects model.

**Supplementary Figure 2:**

**
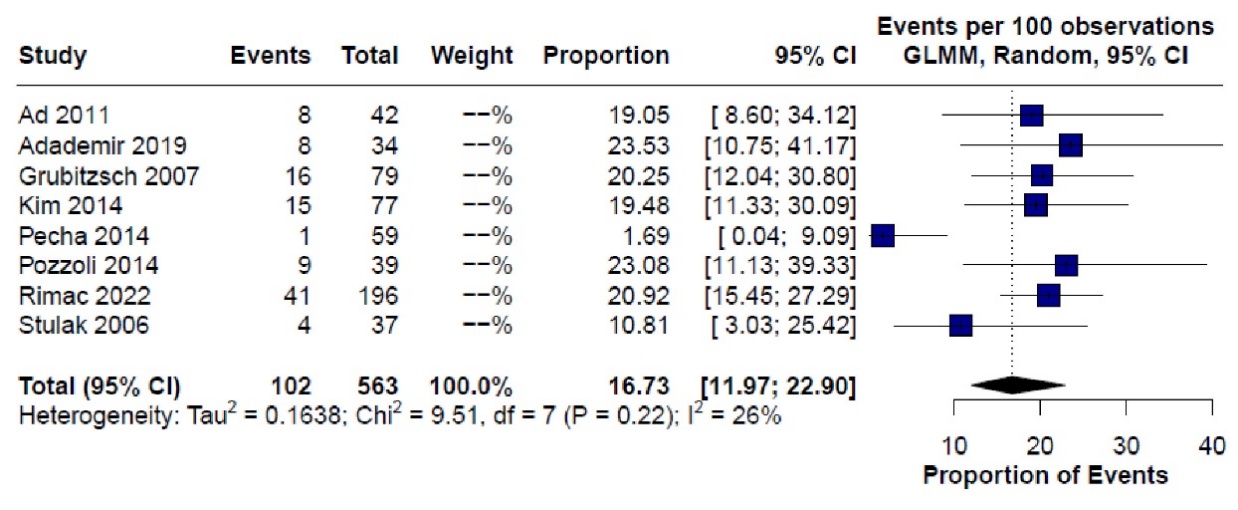
**

Rate of major surgical complications, random effects model. Forest plot of the aggregate evidence showed that complications occurred for 16.73% of patients. CI: confidence interval. GLMM: generalized linear mixed-effects model.

**Supplementary Figure 3:**

**
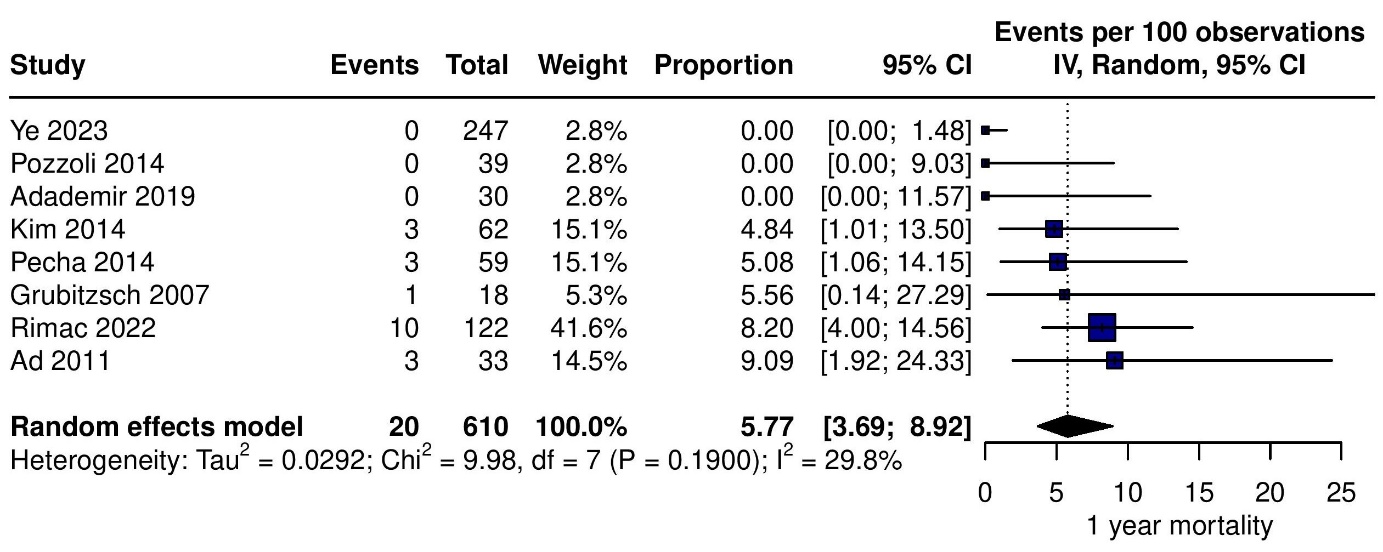
**

Mortality at 1-year, random effects model. Forest plot of the aggregate evidence showing that mortality was 5.8%. CI: confidence interval. GLMM: generalized linear mixed-effects model.

**Supplementary Figure 4:**

**
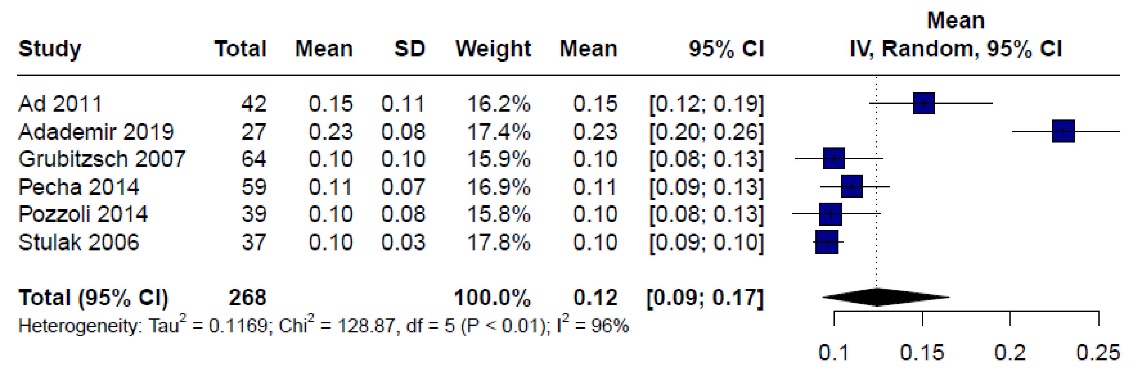
**

Change in LVEF, random effects model. Forest plot of the aggregate evidence showing that LVEF improved by a mean of 12%. CI: Confidence interval. IV: inverse variance method.

**Appendix 3: Surgical characteristics**

**Supplemental table 1. Surgical characteristics of included studies**

| **Study** | **Type of surgery** | **Lesion set** | **Thoracoscopic, %** | **Sternotomy, %** | **CPB time, (mins)** | **ACC time (mins)** | **Energy source** |
| --- | --- | --- | --- | --- | --- | --- | --- |
| Ye 2023 | Concomitant - Valve | CMP IV | 0 | 100 | 108.1±43.7 | 71.9±32.8 | Bipolar RFA |
| Ad 2011 | Standalone ablation or Concomitant – Valve and CABG | CMP III /CMP IV | 0 | 100 | NA | NA | CA or bipolar RFA |
| Grubitzsch 2007 | Concomitant – Valve and CABG | Left atrial ablation with posterior wall isolation. | 0 | 100 | 116±32 | 84±29.2 | MWA or RFA |
| Kim 2014 | Concomitant – Valve and CABG | CMP IV | 0 | 100 | NA | NA | CA or MWA |
| Pecha 2014 | Concomitant – Valve and CABG | Left atrial CMP (51%)/Biatrial CMP (22%)/PVI (27%) | NA | NA | NA | NA | CA or unipolar/bipolar RFA |
| Pozzoli 2014 | Standalone ablation | CMP III (87.2%)  CMP III omitting intercaval line (12.8%) | 56 | 44 | 110±24 | 76 ± 23 | Bipolar RFA or CA |
| Xie 2023 | Standalone ablation | Epicardial ablation – Dalleas set: PVI (39.6%)  PVI+ Linear Ablation (66.4%) | 100 | 0 | NA | NA | Bipolar RFA |
| Stulak 2006 | Standalone ablation or Concomitant - CABG | CMP III with additional cryolesions | 0 | 100 | 117.4±7.3 | 57.1±7.3 | CA |
| Rimac 2022 | Concomitant – Valve and CABG | Biatrial CMP (10.7%) /Left atrial CMP (53.3%)/PVI (36%) | NA | NA | NA | NA | CA and RFA |
| Adademir 2019 | Standalone ablation | Biatrial CMP IV | 26 | 74 | 143±33 | 45±19 | CA and bipolar RFA |
| Mean | - | - | 22.75% | 77.25% | 119.6 | 66.8 | - |

^†^mean or median; CABG: coronary artery bypass grafting; NA: not available; CMP: cox-maze procedure; PVI: pulmonary vein isolation; RFA: radiofrequency ablation; CA: cryoablation; MWA: microwave ablation; ACC: aortic cross clamp; CPB: cardiopulmonary bypass; PVI: pulmonary vein isolation.

**Appendix 4: Sensitivity analysis**

**Supplementary Figure 5:**


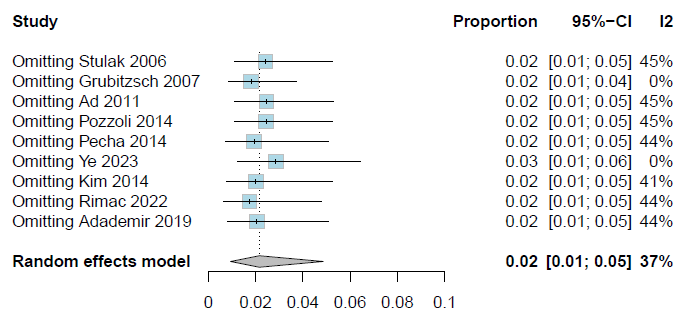


Leave-one-out sensitivity analysis for the outcome of 30-day mortality.

**Supplementary Figure 6:**


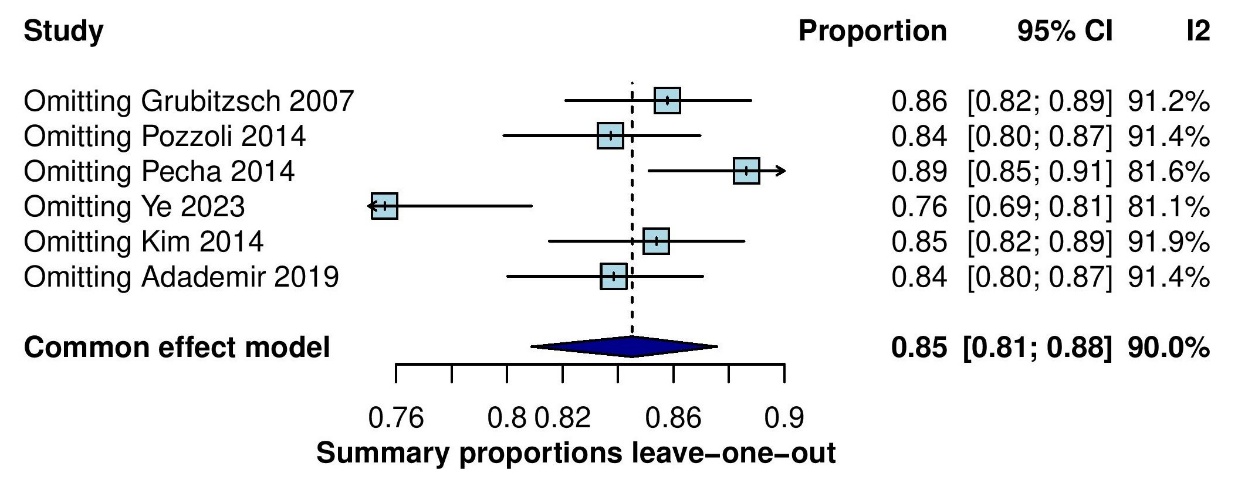


Leave-one-out sensitivity analysis for the outcome of maintenance of sinus rhythm at 1 year.

**Supplementary Figure 7:**


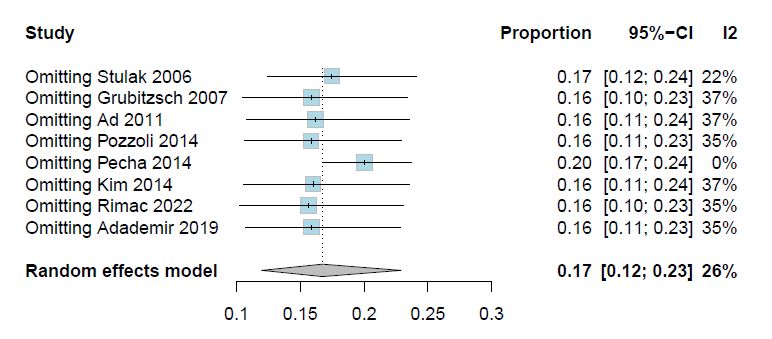


Leave-one-out sensitivity analysis for the outcome of complication rates.

**Supplementary Figure 8:**


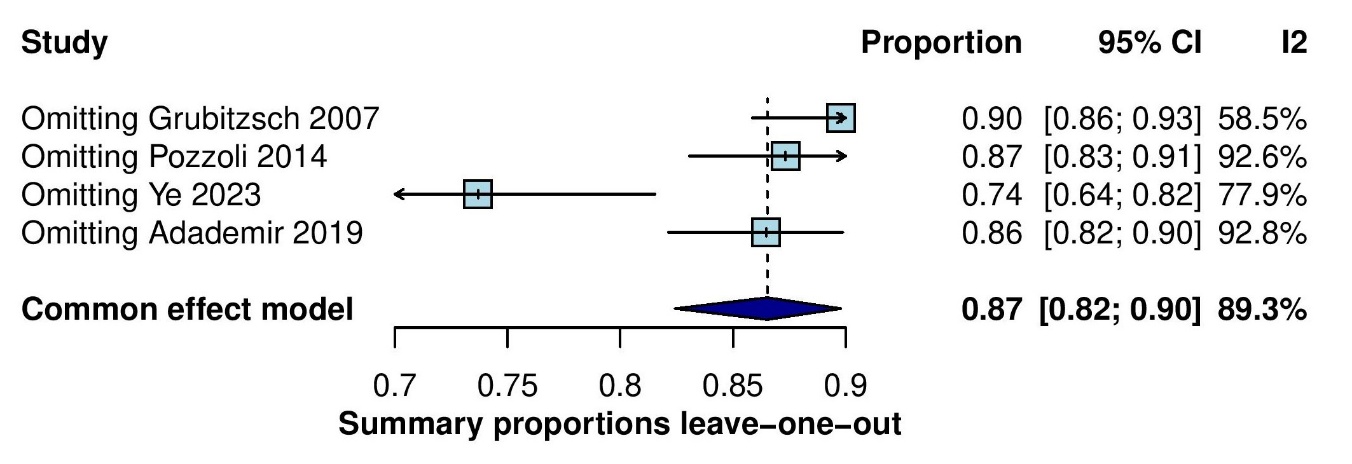


Leave-one-out sensitivity analysis for the outcome of freedom from anti-arrhythmic drug use at 1 year.

**Supplementary Figure 9:**


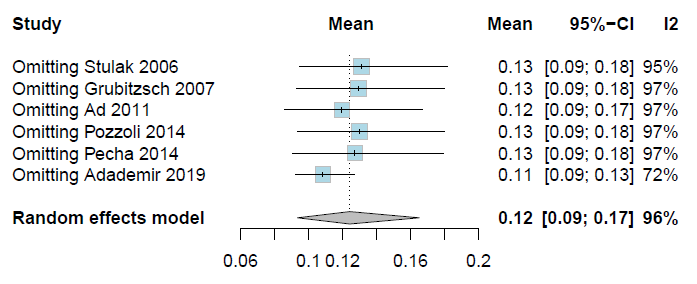


Leave-one-out sensitivity analysis for the outcome of change in left ventricle ejection fraction.

**Supplementary Figure 10:**


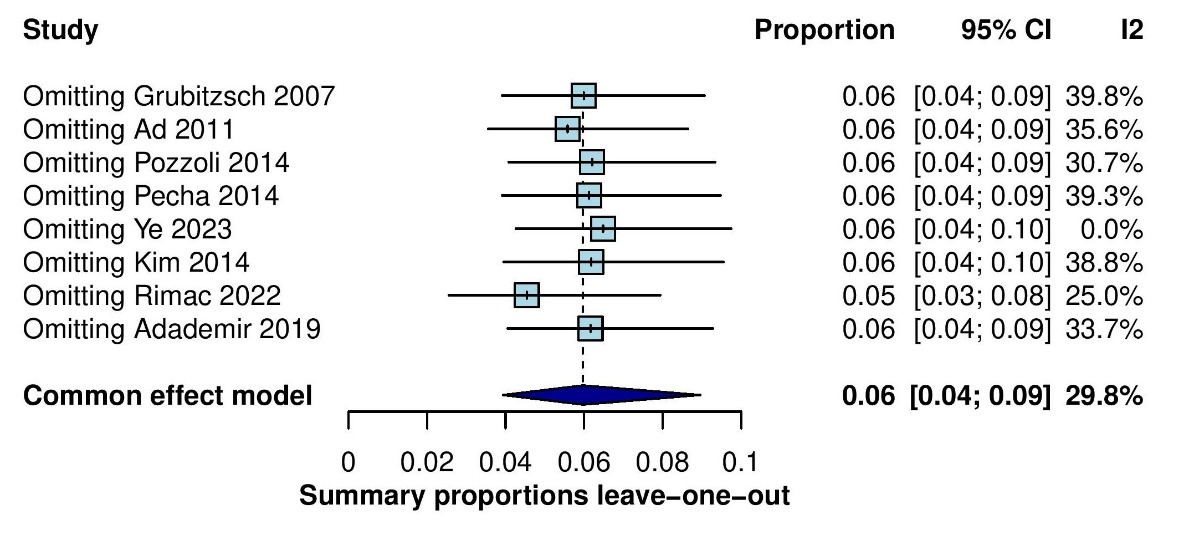


Leave-one-out sensitivity analysis for the outcome of 1-year mortality.

**Supplementary Figure 11:**


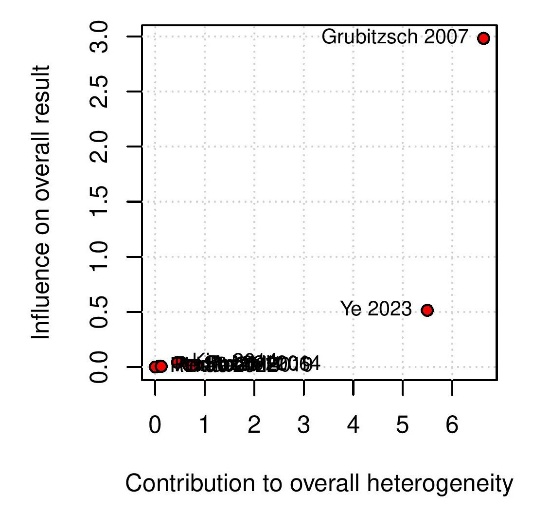


Baujat plot for the outcome of 30-day mortality.

**Supplementary Figure 12:**


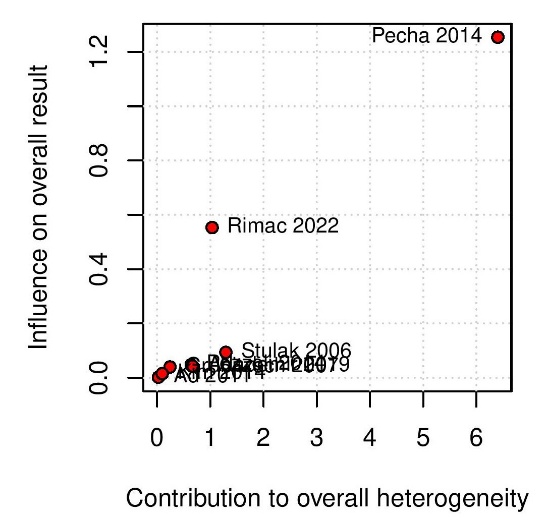


Baujat plot for the outcome of complication rate.

**Supplementary Figure 13:**

**
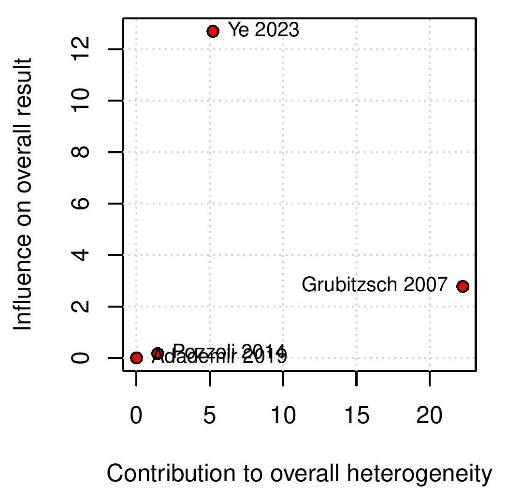
**

Baujat plot for the outcome of freedom from anti-arrhythmic drug use at 1 year.

**Supplementary Figure 14:**


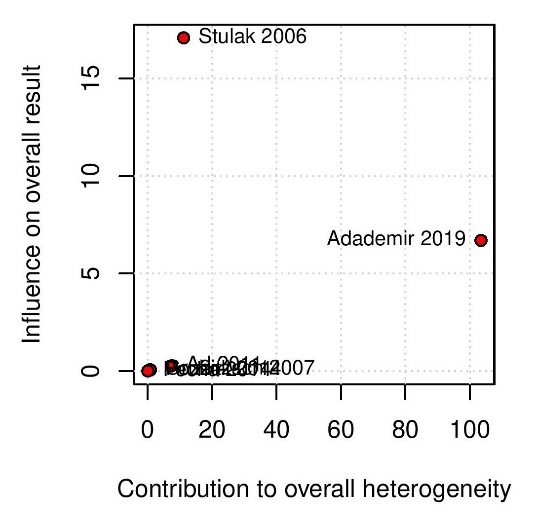


Baujat plot for the outcome of change in left ventricle ejection fraction.

**Supplementary Figure 15:**


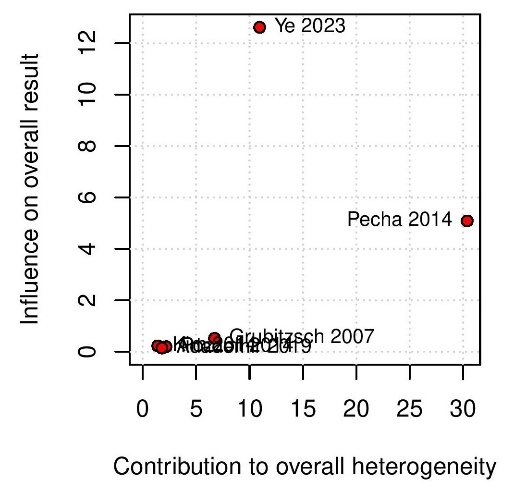


Baujat plot for the outcome of maintenance of sinus rhythm at 1 year.

**Supplementary Figure 16:**


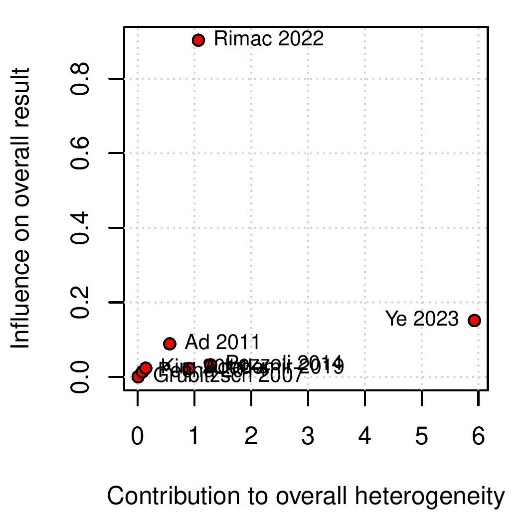


Baujat plot for the outcome of 1-year mortality.

**Supplementary Figure 17:**


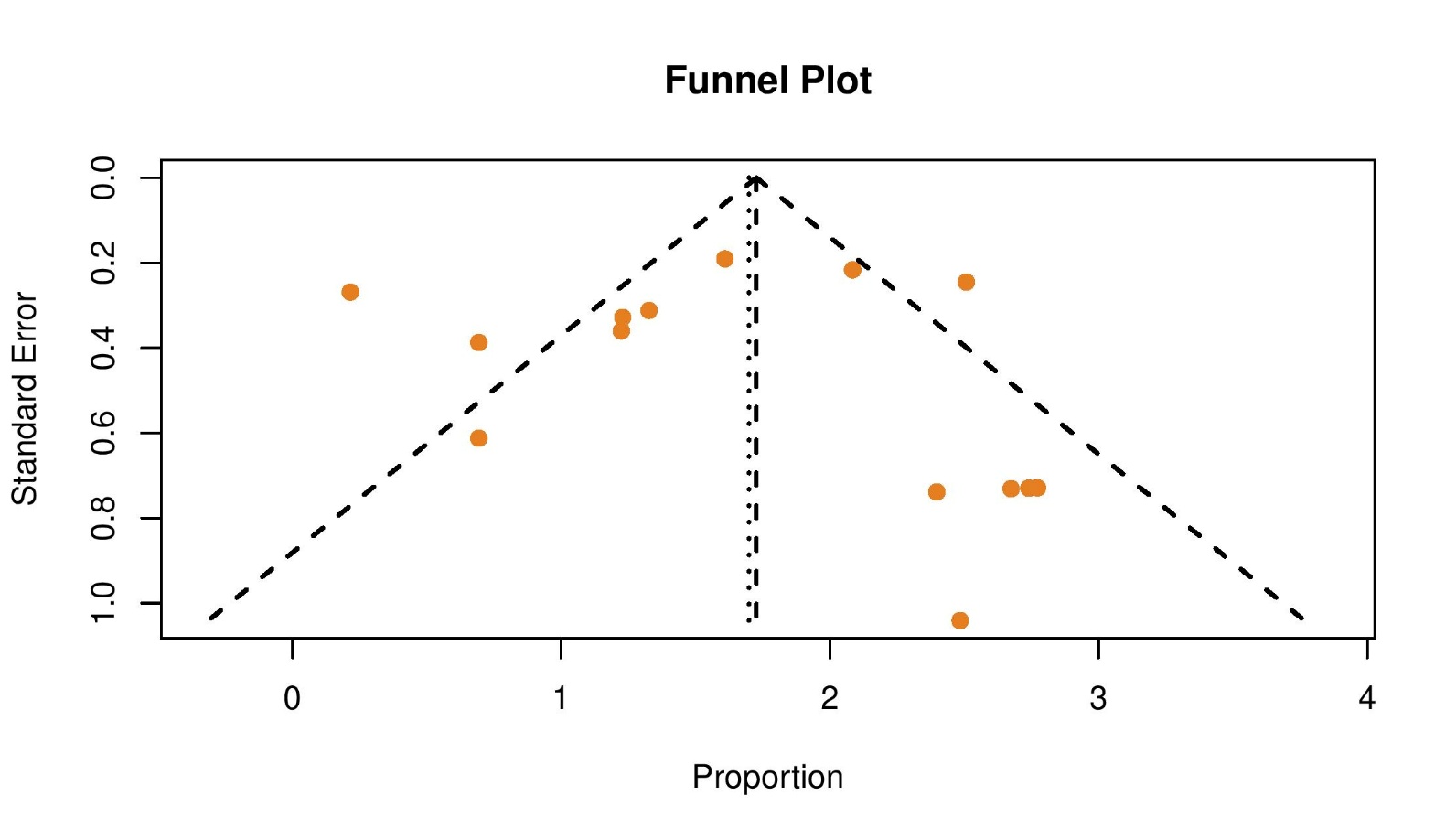


Funnel plot analysis of the studies included in the quantitative synthesis demonstrating significant asymmetry. This result signals a risk of publication bias.

**Supplemental Table 2**: Sensitivity analysis for Pearson’s coefficient

| Coefficient | Effect Size and CI | I^2^ heterogeneity (%) |
| --- | --- | --- |
| 0.1 | 0.1245 [0.0934; 0.1660] | 93.1 |
| 0.2 | 0.1244 [0.0934; 0.1658] | 93.9 |
| 0.3 | 0.1244 [0.0934; 0.1656] | 94.7 |
| 0.4 | 0.1243 [0.0934; 0.1654] | 95.4 |
| 0.5 | 0.1243 [0.0935; 0.1652] | 96.1 |
| 0.6 | 0.1242 [0.0935; 0.1650] | 96.9 |
| 0.7 | 0.1242 [0.0935; 0.1649] | 97.6 |
| 0.8 | 0.1241 [0.0935; 0.1646] | 98.4 |
| 0.9 | 0.1240 [0.0936; 0.1644] | 99.2 |
| 1.0 | 0.1086 [0.0932; 0.1265] | 96.4 |

Legends: CI: confidence interval.

**Appendix 5: Subgroup analysis**

**Supplementary Figure 18:**


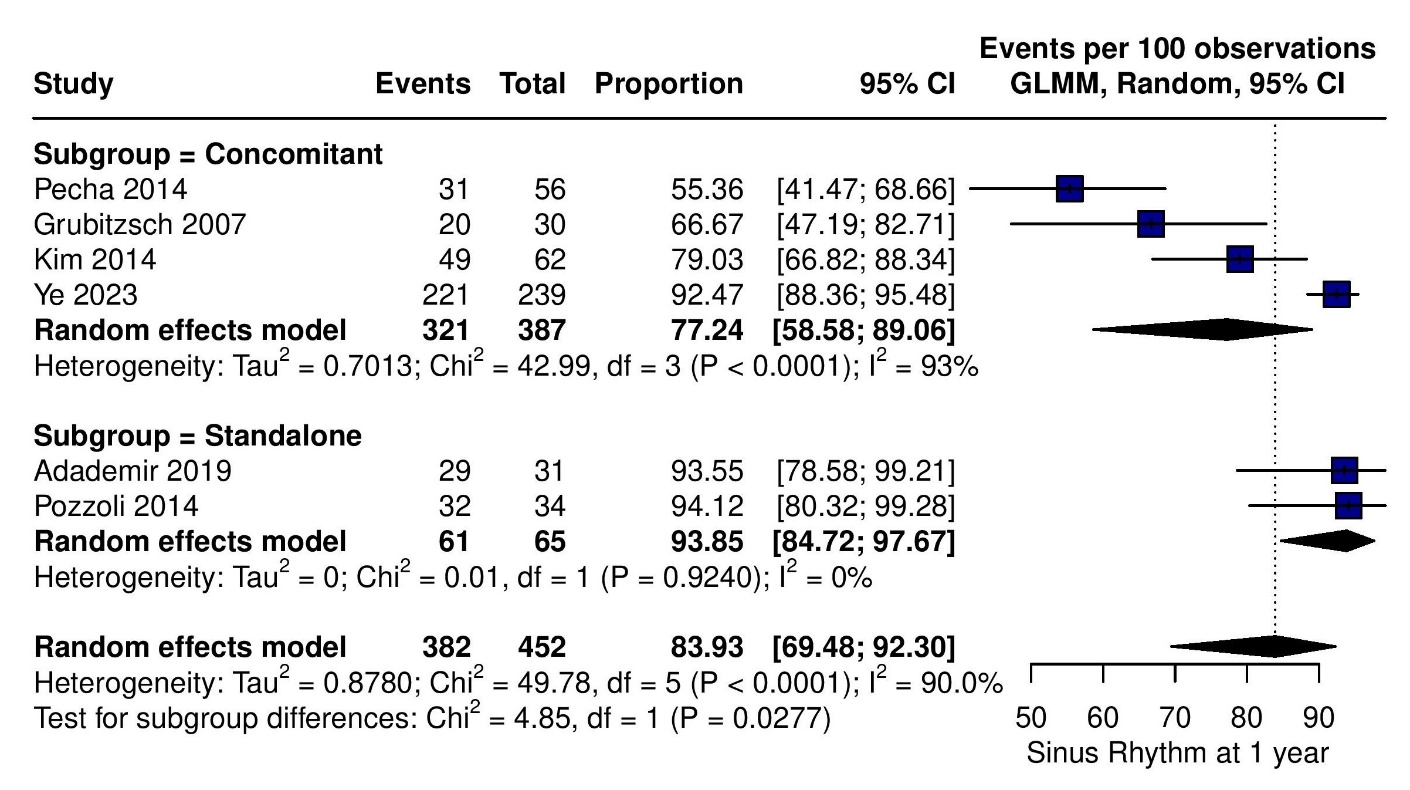


Subgroup analysis for the outcome of sinus rhythm at 1 year comparing standalone and concomitant ablation.

**Supplementary Figure 19:**

**
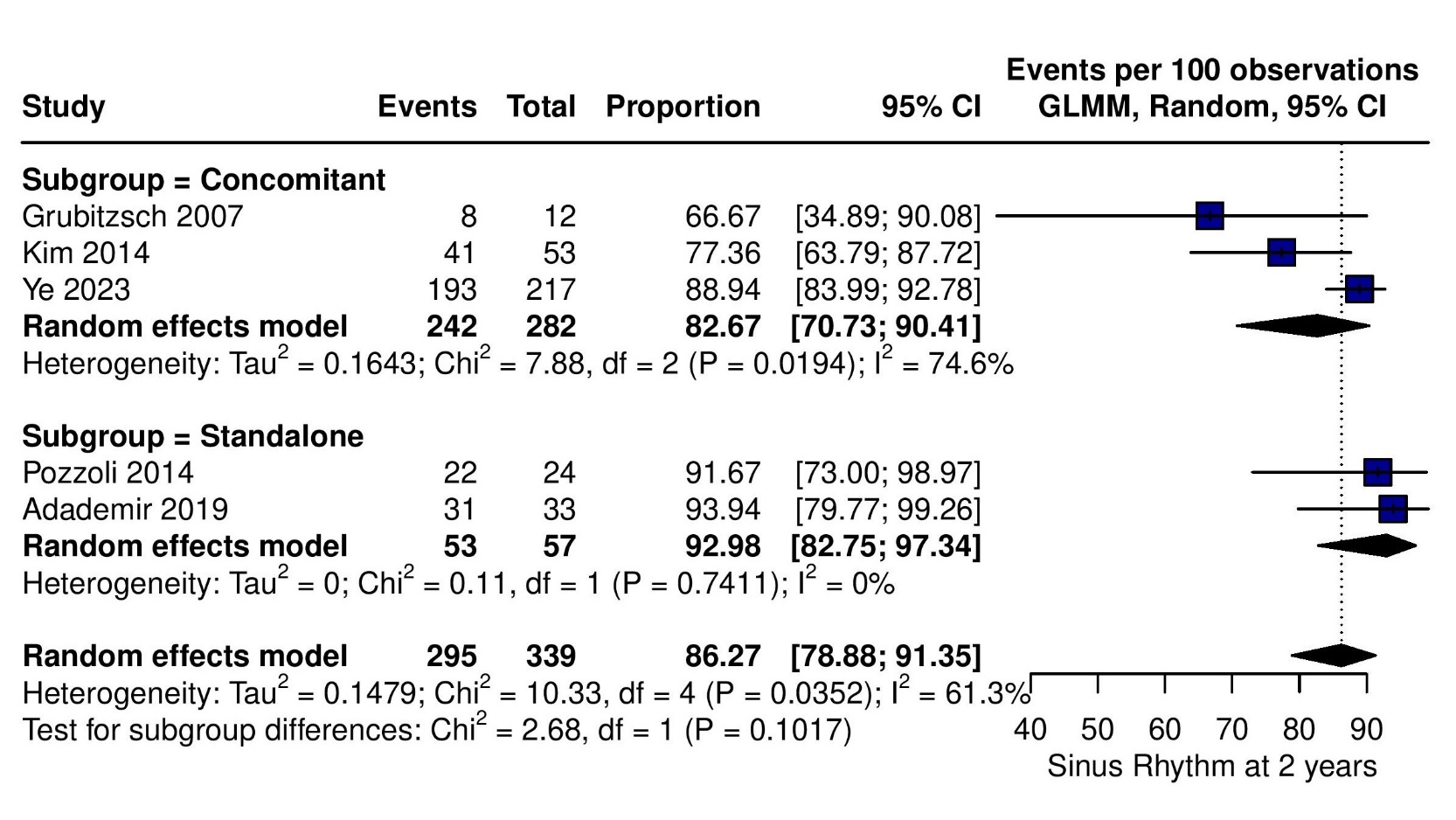
**

Subgroup analysis for the outcome of sinus rhythm at 2 years comparing standalone and concomitant ablation.

**Supplementary Figure 20:**

**
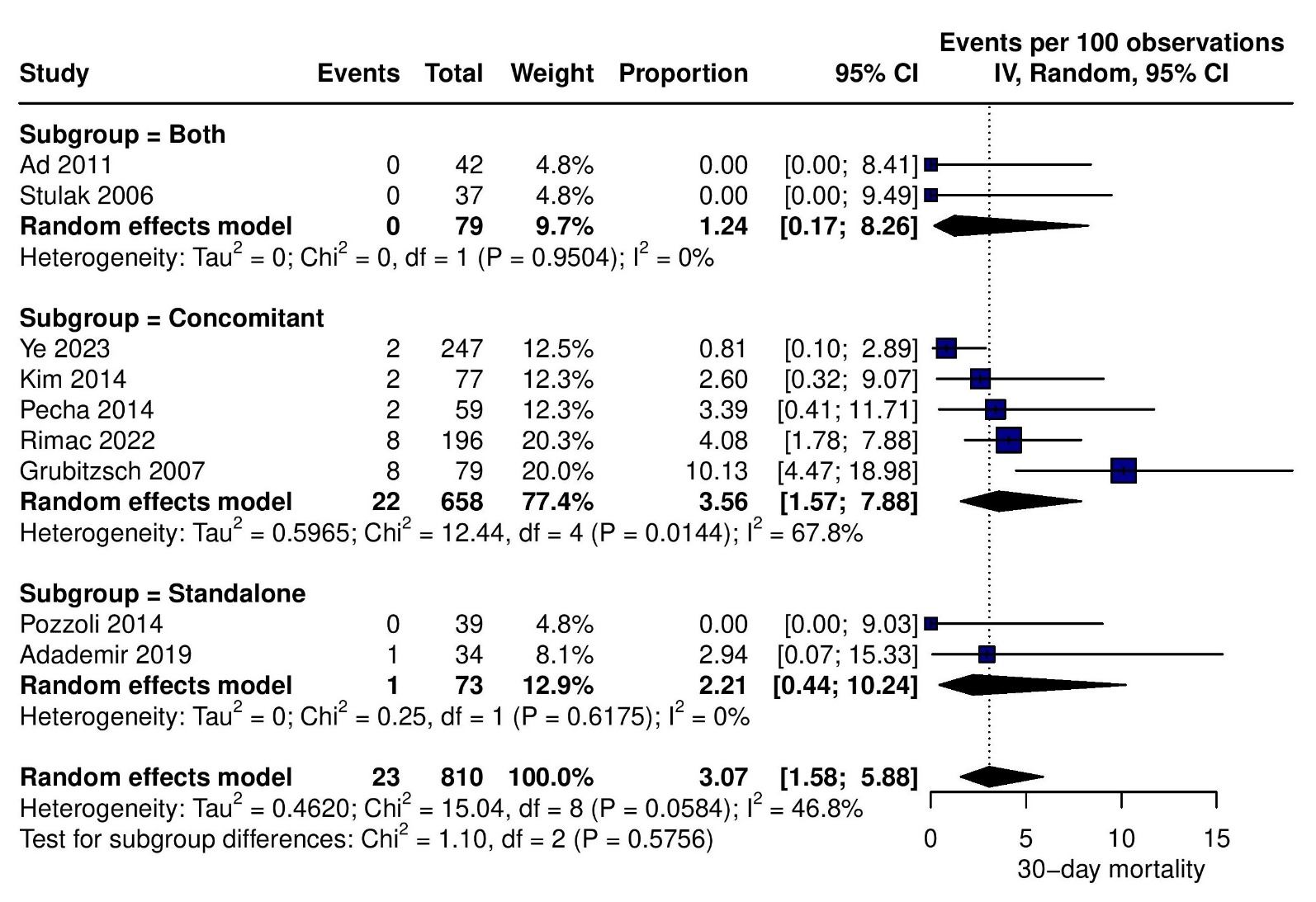
**

Subgroup analysis for the outcome of 30-day mortality comparing standalone and concomitant ablation.

**Supplementary Figure 21:**

**
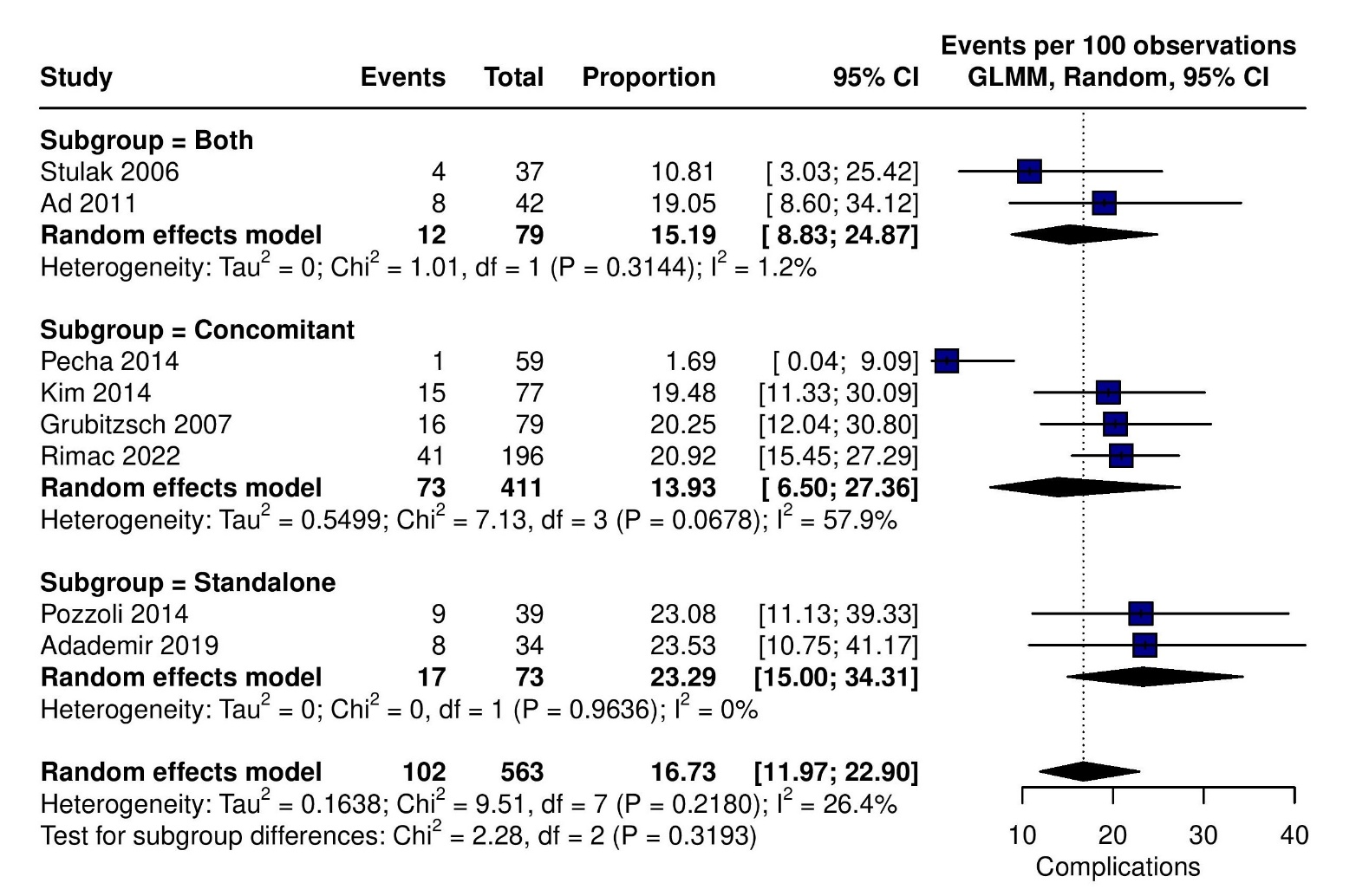
**

Subgroup analysis for the outcome of complications comparing standalone and concomitant ablation.

**Supplementary Figure 22:**

**
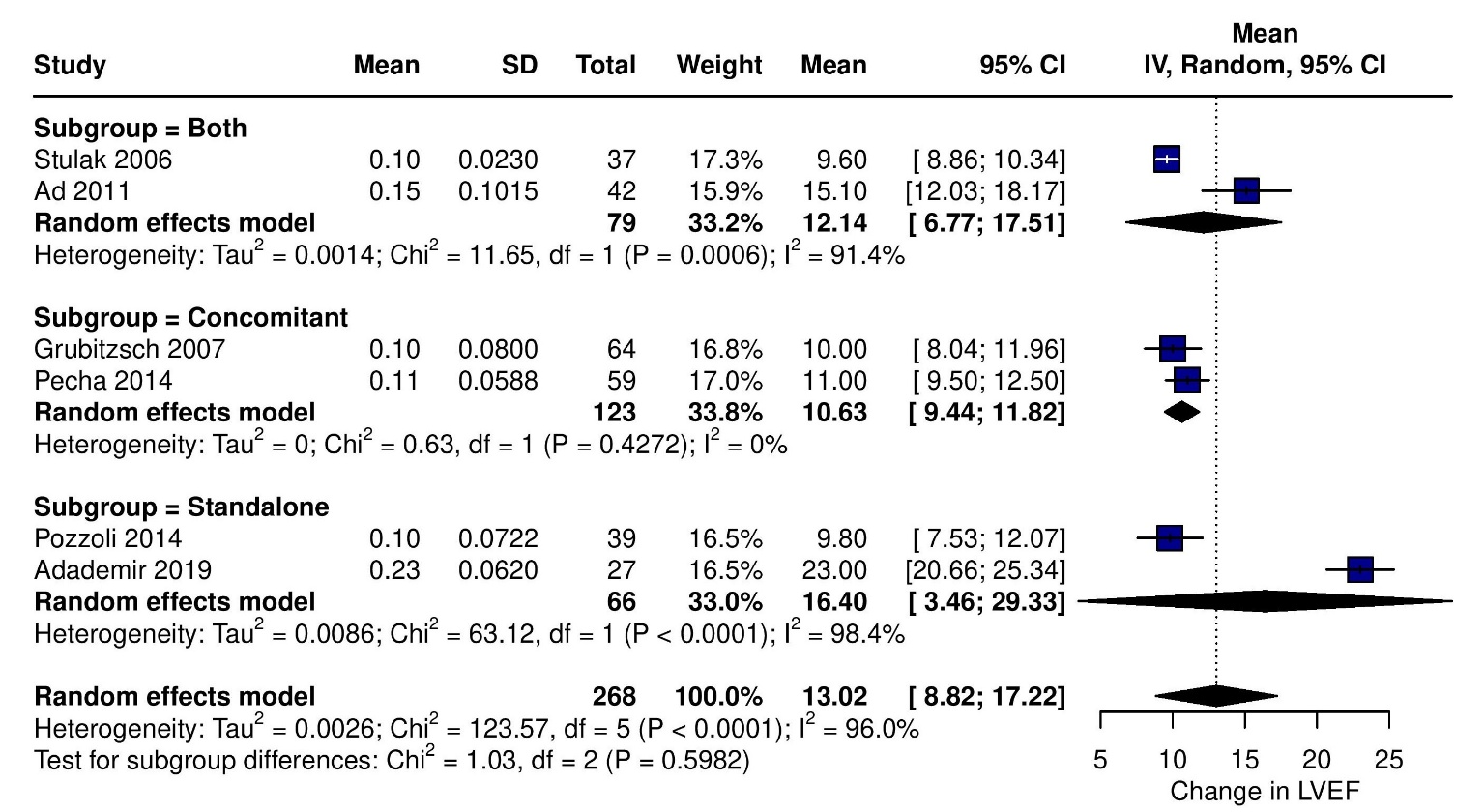
**

Subgroup analysis for the outcome of change in LVEF comparing standalone and concomitant ablation.

**Supplementary Figure 23:**

**
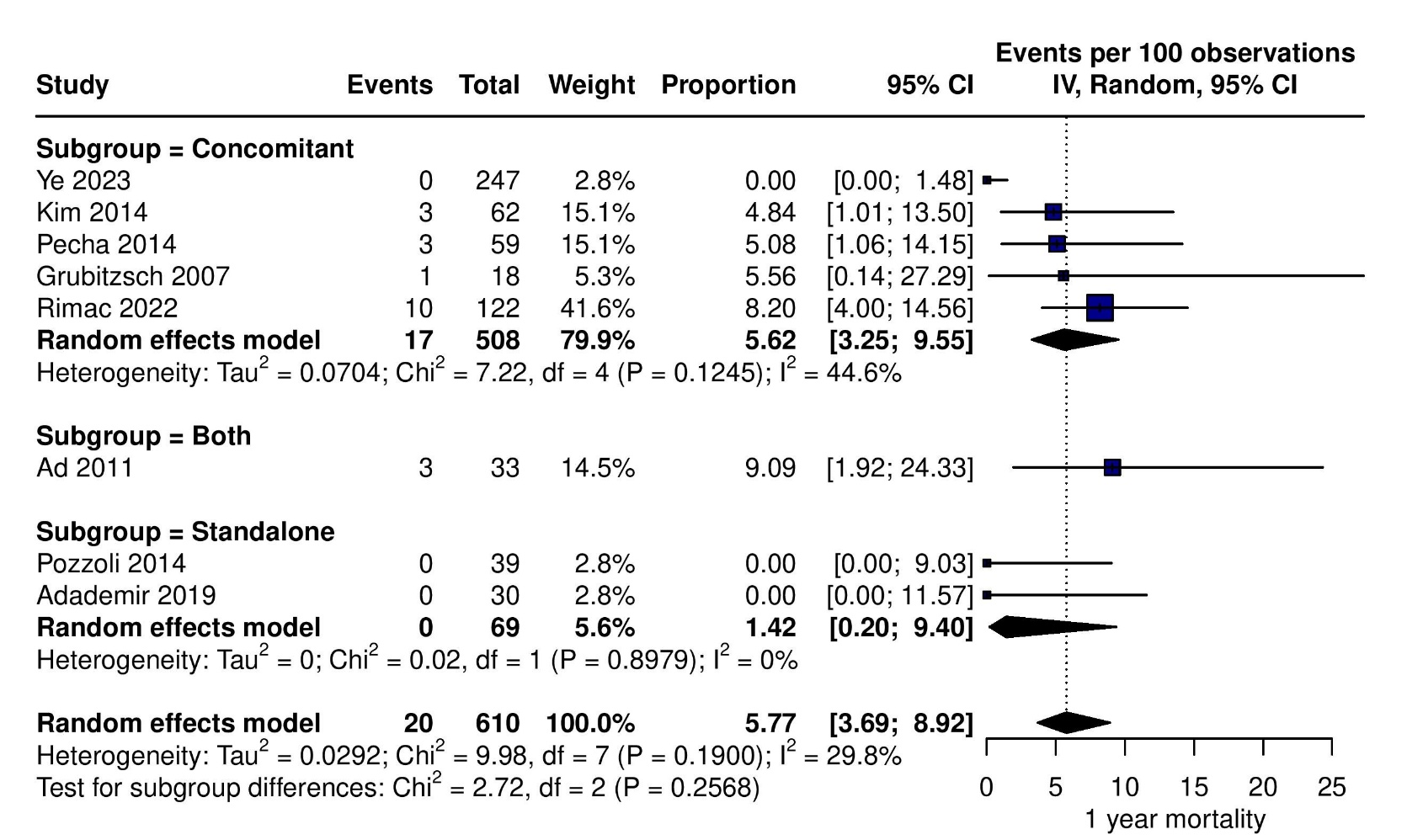
**

Subgroup analysis for the outcome of 1-year mortality comparing standalone and concomitant ablation.

**Supplementary Figure 24:**

**
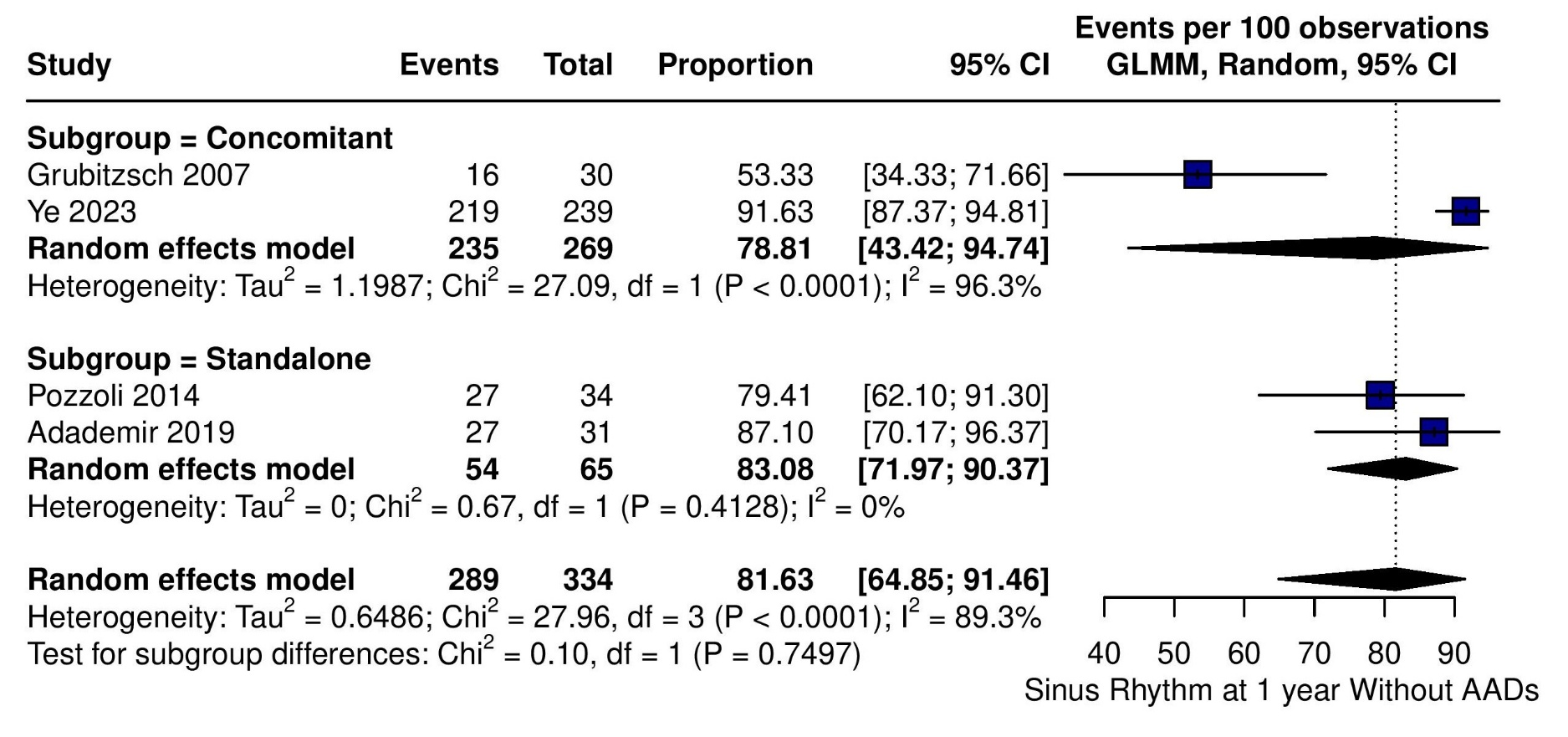
**

Subgroup analysis for the outcome of sinus rhythm without AAD use at 1 year comparing standalone and concomitant ablation.

**Appendix 6: Risk of bias analysis**

**Supplementary Table 3: Risk of bias summary for non-randomized studies (MINORS)**

| **Study** | **Clear stated Aim** | **Inclusion of consecutive patients** | **Prospective collection of data** | **Appropriate endpoints for the aim** | **Unbiased assessment**  **of the endpoints** | **Appropriate follow-up period** | **Follow-up loss < 5%** | **Prospective calculation of sample size** | **Adequate control group** | **Contemporary control group** | **Baseline equivalent groups** | **Adequate statistical analysis** | **Overall risk of bias** |
| --- | --- | --- | --- | --- | --- | --- | --- | --- | --- | --- | --- | --- | --- |
| Pecha | 2 | 2 | 1 | 2 | 1 | 2 | 2 | 0 | - | - | - | - | 12 |
| Stulak | 2 | 2 | 1 | 2 | 1 | 2 | 2 | 0 | - | - | - | - | 12 |
| Kim | 2 | 2 | 1 | 2 | 1 | 2 | 2 | 1 | 2 | 2 | 2 | 2 | 21 |
| Pozzoli | 2 | 1 | 1 | 2 | 1 | 2 | 2 | 0 | - | - | - | - | 11 |
| Grubitzsch | 2 | 2 | 1 | 2 | 1 | 2 | 2 | 0 | 2 | 2 | 0 | 2 | 18 |
| Xie | 2 | 2 | 2 | 2 | 2 | 2 | 2 | 1 | 2 | 2 | 2 | 2 | 23 |
| Ye | 2 | 1 | 2 | 2 | 1 | 2 | 2 | 0 | - | - | - | - | 12 |
| Ad | 2 | 2 | 2 | 2 | 1 | 2 | 2 | 0 | - | - | - | - | 13 |
| Adademir | 2 | 1 | 1 | 2 | 1 | 2 | 2 | 0 | - | - | - | - | 11 |
| Rimac | 2 | 2 | 2 | 2 | 1 | 2 | 2 | 1 | 2 | 2 | 2 | 2 | 22 |
